# Bioinformatics Analysis and Functional Verification of Phytoene Synthase Gene *Pj*PSY1 of *Panax japonicus* C. A. Meyer

**DOI:** 10.3390/cimb47070551

**Published:** 2025-07-16

**Authors:** Tingting Tang, Rui Jin, Xilun Huang, E Liang, Lai Zhang

**Affiliations:** 1 School of Life Science, Guizhou Normal University, Guiyang 550025, China; tang15329537342@163.com (T.T.); jin18786912487@163.com (R.J.); huang222100100420@gznu.edu.cn (X.H.); 2 Innovation Center for Efficient Agricultural of Guizhou Mountain Characteristics, Anshun University, Anshun 561000, China; liang18334110983@163.com

**Keywords:** *Panax japonicus*, Phytoene synthase, carotenoid, bioinformatics, functional validation

## Abstract

Phytoene synthase (PSY) is a multimeric enzyme that serves as the first enzyme in carotenoid synthesis within plant tissues and plays a crucial role in the production of carotenoids in plants. To understand the function of the PSY gene in *Panax japonicus* C. A. Meyer. fruit, the gene’s transcript was obtained by analyzing the transcriptome sequencing data of *Panax japonicus* fruit. The CDS sequence of the gene was cloned from *Panax japonicus* fruit using the RT-PCR cloning technique and named *Pj*PSY1, which was then subjected to biosynthetic analysis and functional verification. The results showed that the open reading frame of the gene was 1269 bp, encoding 423 amino acids, with a protein molecular mass of 47,654.67 KDa and an isoelectric point (pI) of 8.63; the protein encoded by these amino acids was hydrophilic and localized in chloroplasts, and its three-dimensional structure was predicted by combining the pymol software to annotate the N site of action and active centre of the protein. Phylogenetic analysis demonstrated that *Pj*PSY1 had the closest affinity to *Dc*PSY from *Daucus carota*. Overexpression of *Pj*PSY1 led to a significant increase in the content of carotenoid-related monomers in *Arabidopsis thaliana*, with Violaxanthin being synthesized in transgenic *Arabidopsis thaliana* but not in wild-type *Arabidopsis thaliana*. The *Pj*PSY1 clone obtained in this study was able to promote carotenoid synthesis in the fruits of *Panax japonicus*, revealing that the mode of action of *Pj*PSY1 in the carotenoid biosynthesis pathway of *Panax japonicus* fruits has a positive regulatory effect.

## 1. Introduction

*Panax japonicus* C. A. Meyer. is a perennial herb belonging to the genus Ginseng in the family Araliaceae, primarily distributed in Hubei, Yunnan, Guizhou, and Sichuan in China, especially in the Enshi area of Hubei. This plant is classified as one of the rare and endangered ‘seven types of Chinese herbs’ in China, with its main medicinal part being the underground rhizome, which possesses anti-inflammatory, anti-ageing, blood sugar-lowering, and other pharmacological activities, making it highly valuable for medicine and health [1]. The primary medicinal constituents include triterpenoid saponins; additionally, amino acids [2], polysaccharides [3], flavonoids [4], vitamins, and volatile oils [5] have also been reported. At present, there are few reports on the colour changes in the fruits of *Panax japonicus*, which are limited to the dynamic characteristics of the fruit pigment content [6], and no studies on the molecular regulation of carotenoids have been reported. In the fruit, carotenoids are the main pigments [7]. This group of fat-soluble pigments, which include lutein, are widely distributed in nature [8,9] and have a variety of biological functions as widely available pigments in plants. In the carotenoid synthesis pathway, phytoene synthase (PSY) is the central enzyme that determines the total amount of carotenoids accumulated in plant tissues and is the most intensively studied carotenoid metabolism enzyme to date [9]. The PSY gene has been identified and isolated in many species such as *Arabidopsis thaliana* [10], rice [11], maize [12], and tomato [13,14,15]. Many other species have been identified and isolated, and their involvement in carotenoid synthesis has been identified. This study cloned the PSY gene, a key enzyme in carotenoid synthesis, from the fruit of *Panax japonicus* to reveal its role in the variation in colour and shape. The gene was analyzed bioinformatically and subsequently overexpressed in *Arabidopsis thaliana* to further verify its function, establishing a foundation for in-depth research on the regulatory mechanisms of carotenoid biosynthesis in *Panax japonicus*.

## 2. Materials and Methods

### 2.1. Plant Materials

The experimental materials were provided by the Innovation Centre for Efficient Agricultural of Guizhou Mountain Characteristics and identified as *Panax japonicus* C. A. Meyer.

### 2.2. Methods

#### 2.2.1. Total RNA Extraction and cDNA Synthesis of *Panax japonicus* Fruits

Fresh fruits of *Panax japonicus* were selected, RNA was extracted using the Plant Total RNA Extraction Kit (Tiangen, Beijing, China), and cDNA was synthesized with the TIAN Script II cDNA First Strand Synthesis Kit (Tiangen). The samples were stored at −20 °C for future use.

#### 2.2.2. Amplification of *Pj*PSY1 Gene

Based on the transcriptome data of *Panax japonicus* fruit, the cDNA of the fruit was used as the template. The PCR reaction system consisted of 12.5 μL of 2× PCR Mix, 1 μL of *Pj*PSY1-F, 1 μL of *Pj*PSY1-R, 2 μL of cDNA, and 8.5 μL of ddH_2_O. The PCR program included pre-denaturation at 95 °C for 5 min, denaturation at 95 °C for 15 s, annealing at 58 °C for 15 s, extension at 72 °C for 30 s, followed by 30 cycles, extension at 72 °C for 8 min, and storage at 4 °C. PCR products were analyzed using 1% agarose gel electrophoresis, and the products were purified and stored at −20 °C for future use. The primer sequences required for the experiment were as follows:

*Pj*PSY1-F: TTGGAGAGAACACGGGGGACTCTAGAATGTGCCTTTGCATATGCAACCGGTAAAGCAAG

*Pj*PSY1-R: GTAACATAAGGGACTGACCACCCGGGTCAATGTCAGTTGCTCTGGTGTGGGTTGTTTCC

#### 2.2.3. Bioinformatics Analysis of the *Pj*PSY1 Gene

Sequencing results were subjected to open reading frame identification and translated into amino acid sequences using SnapGene6.0.2. The protein sequences were analyzed for conserved domains, physicochemical properties, and amino acid hydrophilicity and hydrophobicity using online resources at https://www.ncbi.nlm.nih.gov/Structure/cdd/wrpsb.cgi, https://web.expasy.org/cgi-bin/protparam/protparam, and https://web.expasy.org/protscale (accessed on 9 March 2025). *Pj*PSY1 interacting protein prediction was performed using STRING12.0. The secondary and tertiary structures of the protein were predicted using the SOPMA (https://npsa-prabi.ibcp.fr/cgi-bin/npsa_automat.pl?page=npsa%20_sopma.html, accessed on 9 March 2025) and SWISS-MODEL (https://www.expasy.org/resources/swiss-model, accessed on 10 March 2025) online tools, and subcellular localization of the *Pj*PSY1 protein was predicted with Cell-PLoc 2.0 (http://www.csbio.sjtu.edu.cn/bioinf/Cell-PLoc-2/, accessed on 10 March 2025). The *Pj*PSY1 protein was compared using BLAST (https://blast.ncbi.nlm.nih.gov/Blast.cgi, accessed on 10 March 2025), and sequences from different families and genera that closely resembled the *Pj*PSY1 protein were selected for phylogenetic tree analysis, using the neighbour-joining algorithm (bootstrap r:1000) with MEGAX64 software.

#### 2.2.4. Construction and Transformation of *Pj*PSY1 Plant Expression Vector

##### Construction of pBI121-*Pj*PSY1 Recombinant Vector

The recombinant plasmid vector pBI121-*Pj*PSY1 was obtained by cleaving the pBI121 vector with XbaI and XmaI, followed by the ligation of pBI121 and *Pj*PSY1 with T4-DNA ligase. The recombinant plasmid vector pBI121-*Pj*PSY1 was introduced into *E. coli* DH5α via heat shock. LB liquid medium was added to the bacteria, which were mixed well and incubated at 37 °C with shaking at 150 rpm for 45 min to express the *Pj*PSY1 gene on the pBI121 plasmid. The transformed cells were plated on LB + 50 mg/L Kan solid medium, inverted, and incubated at 37 °C for 12–16 h. Individual colonies were picked for positive screening, and positive strains were preserved in 50% glycerol at −80 °C.

##### Transformation of *Arabidopsis thaliana* with pBI121-*Pj*PSY1 Recombinant Vector

The recombinant plasmid vector pBI121-*Pj*PSY1 was transferred to *Agrobacterium tumefaciens* GV3101 using the freeze–thaw method, mixed with 890 μL of YEP liquid medium, and incubated in the dark at 28 °C at 220 rpm for 3–4 h. The bacterial solution was centrifuged at 4 °C at 6000 rpm for 1 min, and the pellet was resuspended in YEP + 25 mg/L Rif + 50 mg/L Kan solid medium and incubated at 28 °C for 2–3 days. After single colonies grew, a colony was inoculated into 2 mL YEP + 50 mg/L Rif + 50 mg/L Kan liquid medium and incubated at 28 °C at 180 rpm for 12–16 h. The bacterial solution underwent PCR identification, and positive bacterial solutions were shaken until OD600 reached 0.8–1.0, then centrifuged to collect the cells. The cells were then resuspended in an aqueous sucrose solution and incubated until OD600 reached 0.5–0.8 for infiltration of *Arabidopsis thaliana* (Columbia-0) inflorescences. Gene expression in transgenic *Arabidopsis thaliana* of T1 and T2 generations was assessed by PCR.

##### Quantitative Fluorescence Analysis and Carotenoid Content of Trans-*Pj*PSY1 *Arabidopsis thaliana*

Total RNA from *Arabidopsis thaliana* plants identified as positive was extracted and reverse-transcribed into cDNA. Using this cDNA as a template and Actin as the internal reference gene, *Pj*PSY1 gene expression analysis for both wild-type and trans-*Pj*PSY1 *Arabidopsis thaliana* was conducted via real-time fluorescence quantification. The 2^−ΔΔCt^ algorithm was utilized to analyze the relative expression level of the *Pj*PSY1 gene. Carotenoid-related compounds in wild-type and trans-*Pj*PSY1 *Arabidopsis thaliana* were analyzed using ultra-performance liquid chromatography (UPLC) [16].

## 3. Results

### 3.1. Successful Cloning of the PjPSY1 Gene of Panax japonicus

Based on the transcriptome data, we successfully cloned the PSY gene of *Panax japonicus*, with an ORF length of 1269 bp, encoding 423 amino acids. The NCBI accession number is PV471061, and the gene is named *Pj*PSY1.

### 3.2. Biological Information About the PjPSY1 Gene

#### 3.2.1. Physicochemical Properties of *Pj*PSY1

The prediction results show that the molecular weight of the *Pj*PSY1 protein is 47,654.67 kDa, the molecular formula is C_2119_H_3364_N_580_O_630_S_19_, the total number of negatively charged amino acids (Asp + Glu) is 53, and the total number of positively charged amino acids (Arg + Lys) is 58. The total number of atoms is 6712, and the aliphatic index is 88.56. Theoretical pI was 8.63, indicating that the protein is basic. The instability index of the protein was 48.6, which suggests that it can be regarded as unstable.

#### 3.2.2. *Pj*PSY1 Conserved Domains

**Figure 1:**
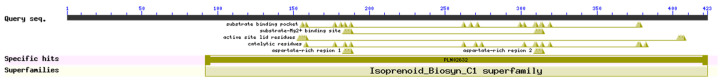
*Pj*PSY1 conserved domain.

Predictions show that *Pj*PSY1 belongs to the superfamily of trans-isoprenyl diphosphate synthases (IPPS) and class I terpene cyclases (Figure 1). Together, they are involved in key steps of the carotenoid biosynthetic pathway and act synergistically at the molecular level. PSY enzyme directly catalyzes the production of Phytoene from GGPP, and its efficient catalysis relies on the availability of sufficient GGPP substrate from IPPS, while the synergistic action of class I terpene cyclases may optimize the balance of upstream metabolic streams, thus ensuring the efficient operation of PSY enzyme.

#### 3.2.3. Amino Acid Hydrophilicity, Signal Peptide and Subcellular Localization

**Figure 2:**
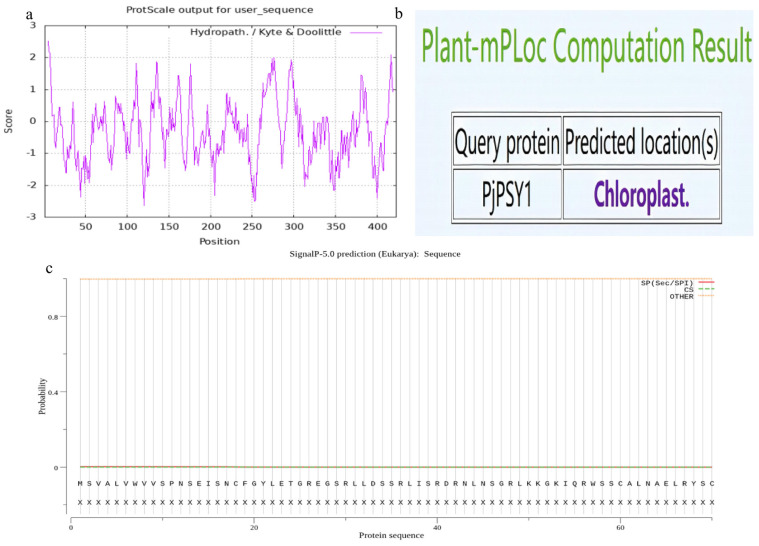
*Pj*PSY1 hydrophobicity, signal peptide, and subcellular localization. Note: (**a**) hydrophobicity of amino acids, (**b**) subcellular localization, (**c**) protein signal peptide.

The results indicated that the strongest hydrophilic amino acid residues of the *Pj*PSY1 protein were located at amino acid position 120 (MIN: −2.633), while the strongest hydrophobic amino acid residues were at amino acid position 5 (MAX: 2.511). As shown in Figure 2a, the grand average of hydropathicity is −0.285, indicating that *Pj*PSY1 is a hydrophilic protein. The *Pj*PSY1 protein does not have a signal peptide (Figure 2c), and it is located in the chloroplasts (Figure 2b).

#### 3.2.4. Protein Secondary Structure and Tertiary Structure

**Figure 3:**
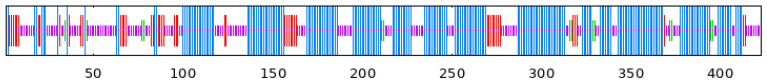
Secondary structure prediction results. Note: blue is α-helix; red is extended strand; green is beta turn; purple is random coil.

**Figure 4:**
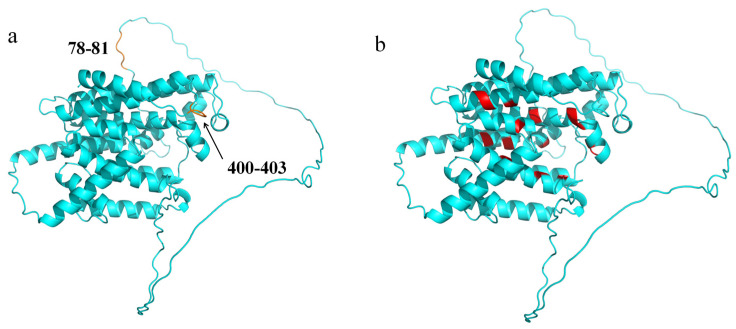
*Pj*PSY1 tertiary structure. Note: (**a**) is the N action site, and (**b**) is the active centre.

The prediction results are shown in Figure 3. The secondary structure of *Pj*PSY1 consists of alpha helices, extended strands, beta turns, and random coils. The prediction of the tertiary structure of *Pj*PSY1 was carried out by the online tool SWISS-MODEL, and the protein with the highest sequence identity to the *Pj*PSY1 protein was selected as a template for homology modelling in the protein database to obtain the structural model of the *Pj*PSY1 protein of *Panax japonicus* (Figure 4). The N site of action and active centre of the protein were annotated in combination with pymol 3.1.0 software, as shown in Figure 4a and Figure 4b, respectively.

#### 3.2.5. *Pj*PSY1 Interacting Proteins

**Figure 5:**
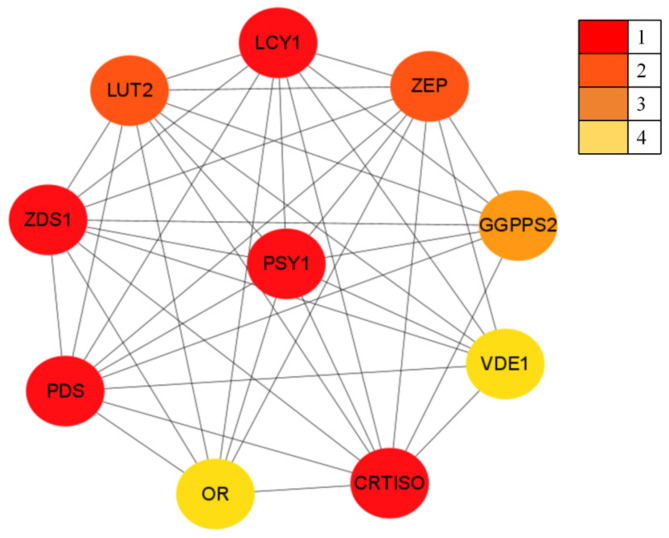
The *Pj*PSY1 protein interaction diagram. Note: 1, 2, 3, and 4 are classified into four classes based on the number of interacting proteins.

In the STRING database, *Pj*PSY1 was matched to the *Arabidopsis thaliana* PSY1 gene. Predictions indicated (Figure 5) that PSY1 has potential interacting proteins, including interactions with phytoene desaturase (Q07356), Prolycopene isomerase, (Q9M9Y8), Zeta-carotene desaturase, (Q38893), Violaxanthin de-epoxidase (Q39249), Lycopene beta cyclase (Q38933), Zeaxanthin epoxidase (Q9FGC7), Heterodimeric geranylgeranyl pyrophosphate synthase (P34802), and Lycopene epsilon cyclase (Q38932), as well as the ORANGE protein (Q9FKF4). Based on the prediction results, it was hypothesized that *Pj*PSY1 has potential interactions with the above proteins.

#### 3.2.6. Evolutionary Tree Analyses

**Figure 6:**
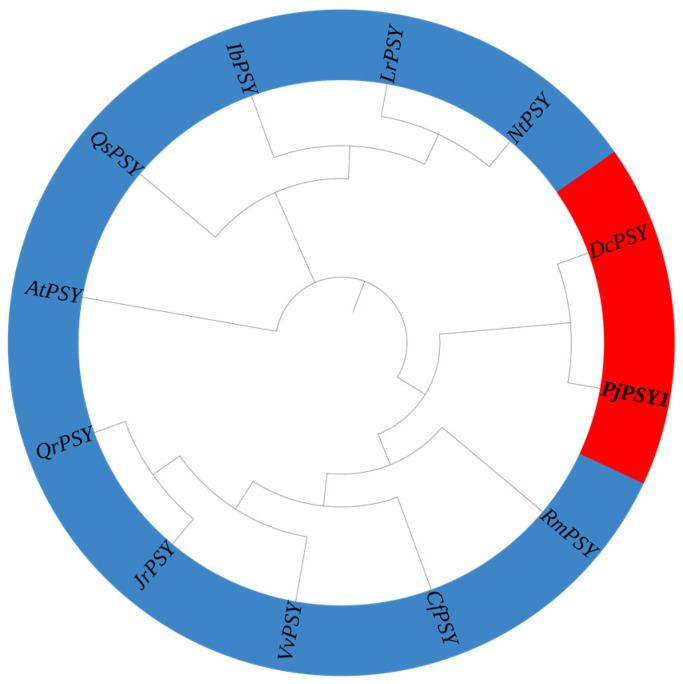
Results of the evolutionary tree analysis.

The homologous sequence of *Pj*PSY1 was obtained by Blast comparison, which compared *Pj*PSY1 of *Panax japonicus* with *Rhododendron molle* (*Rm*PSY, APB08593.1), *Nicotiana tabacum* (*Nt*PSY, ADZ24219.1), *Daucus carota* (*Dc*PSY, NP_001316096.1), *Cornus florida* (*Cf*PSY, XP_059645444.1), *Juglans regia* (*Jr*PSY, XP_018816875.1), *Quercus robur* (*Qr*PSY, XP_050270375.1), *Quercus suber* (*Qs*PSY, XP_023872284.1), *Vitis vinifera* (*Vv*PSY, XP_002271575.1), *Lycium ruthenicum* (*Lr*PSY, AIX87518.1), *Ipomoea batatas* (*Ib*PSY, AGL44391.1), and *Arabidopsis thaliana* (*At*PSY, NP_197225.1). The amino acid sequences of the 11 plant PSY genes were used to construct an evolutionary tree. The results (Figure 6) showed that *Pj*PSY1 was the closest relative to *Daucus carota* (*Dc*PSY, >NP_001316096.1), indicating that the *Pj*PSY1 protein of *Panax japonicus* has a similar function to the *Dc*PSY protein.

### 3.3. PjPSY1 Gene Function

#### 3.3.1. Successful Construction of the pBI121-*Pj*PSY1 Recombinant Vector

**Figure 7:**
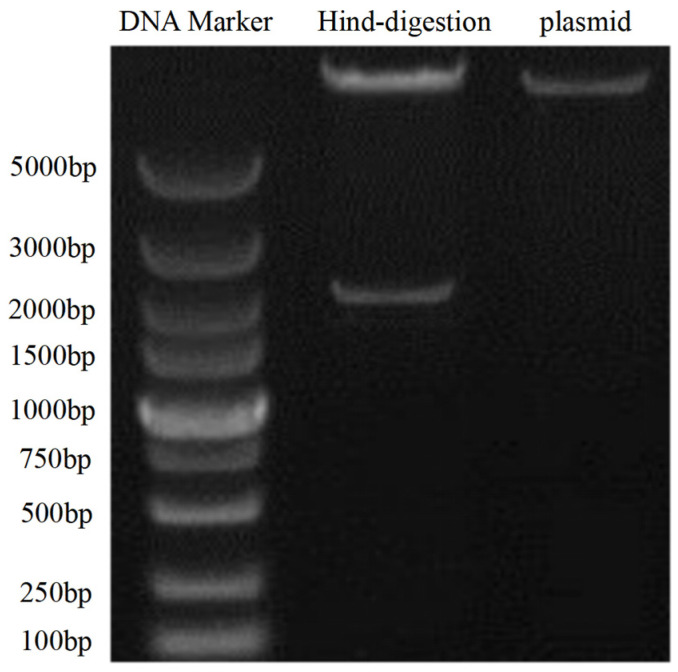
Digestion verification of pBI121-*Pj*PSY1.

The results of digestion verification are shown in Figure 7. Using the plasmid DNA bands as a control, the HindIII digestion results display two bands of pBI121 plasmid DNA and *Pj*PSY1 DNA, indicating that the pBI121-*Pj*PSY1 recombinant vector was successfully constructed.

#### 3.3.2. Successful Acquisition of Trans-*Pj*PSY1 *Arabidopsis thaliana*

**Figure 8:**
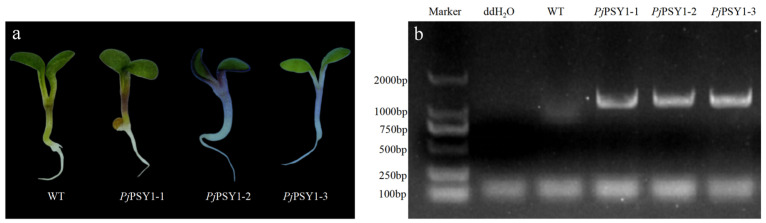
Transfer to *Pj*PSY1 *Arabidopsis thaliana* phenotype and *Arabidopsis thaliana* PCR identification. Note: (**a**) *Arabidopsis thaliana* phenotype; (**b**) PCR identification.

*Pj*PSY1 was expressed in *Arabidopsis thaliana* using the flower dip method, and the seeds obtained were screened for germination on Kan-supplemented medium, resulting in three positive plants. Phenotypic identification revealed that the stalks and leaves of the positive plants exhibited a purple or purplish-red colour (Figure 8a). The positive *Arabidopsis thaliana* seedlings were transferred to soil for incubation, and their DNA was extracted for PCR identification, using water and wild-type *Arabidopsis thaliana* as controls. The results indicated that *Pj*PSY1 was amplified in the three screened *Arabidopsis thaliana* seedlings, while no amplification occurred in the water or wild type, confirming that trans-*Pj*PSY1 *Arabidopsis thaliana* was successfully obtained (Figure 8b).

#### 3.3.3. Correlation Between *Pj*PSY1 Expression and Carotenoid Synthesis

**Figure 9:**
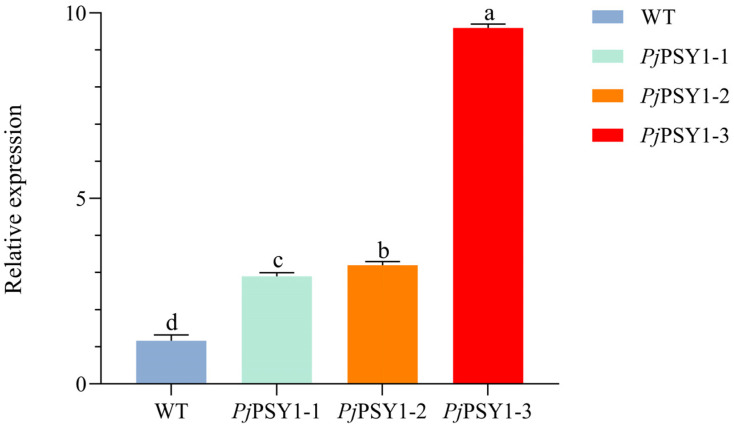
Quantitative analysis of the *Pj*PSY1 fluorescence. Note: Different letters represent different variability (*p* < 0.05).

**Figure 10:**
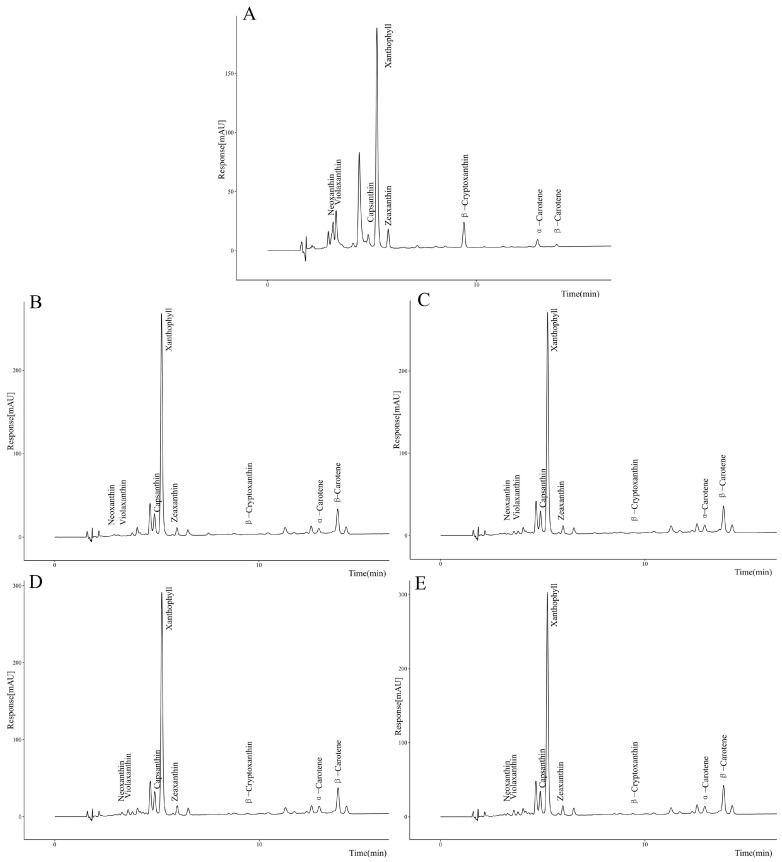
The liquid phase map of the carotenoids. Note: (**A**) is a standard, (**B**) is WT, (**C**) is *Pj*PSY1-1, (**D**) is *Pj*PSY1-2, and (**E**) is *Pj*PSY1-3.

**Figure 11:**
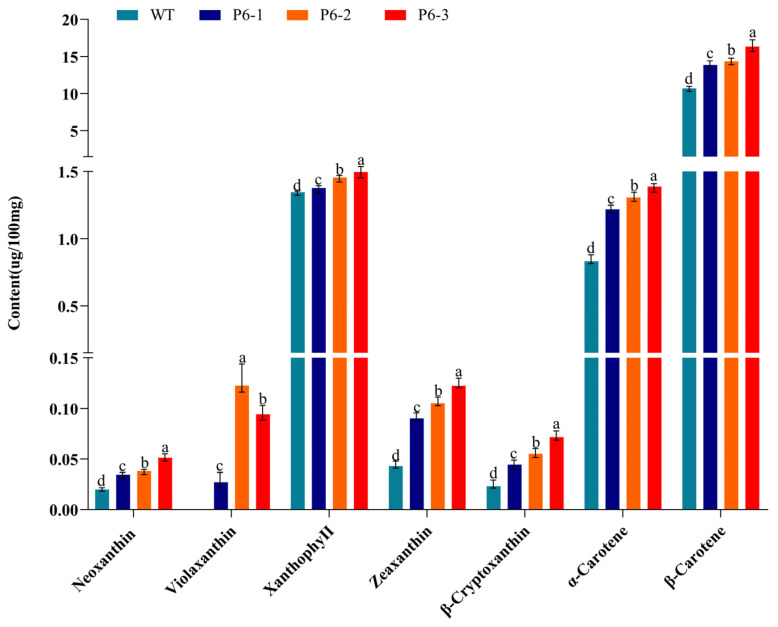
Content of different carotenoid monomers. Note: Different letters represent different variability (*p* < 0.05).

To verify whether *Pj*PSY1 overexpression promotes carotenoid biosynthesis, trans-*Pj*PSY1 *Arabidopsis thaliana* was produced using the flower dip method, and positive plants were identified by PCR. The expression of *Pj*PSY1 was quantitatively analyzed via fluorescence, with the wild type serving as a control, and its carotenoid content was also measured. The results (Figure 9) demonstrated that the expression of *Pj*PSY1 in trans-*Pj*PSY1-positive *Arabidopsis thaliana* was significantly higher than that in wild-type *Arabidopsis thaliana*. Furthermore, the HPLC assay (Figure 10) determined the following carotenoid levels: Neoxanthin, Violaxanthin, Xanthophyll, Zeaxanthin, β-Cryptoxanthin, α-Carotene, and β-Carotene. The contents of each carotenoid monomer in the three transgenic *Pj*PSY1 lines were higher than those in the wild type (Figure 11). Additionally, Violaxanthin was synthesized in the transgenic lines but was undetectable in wild-type *Arabidopsis thaliana*. Combined with the key role of Violaxanthin as a light-responsive molecule, it is predicted that this gene may enhance the ability of *Arabidopsis thaliana* to perceive and adapt to the light environment. Therefore, it can be concluded that the cloned *Pj*PSY1 gene promotes an increase in carotenoid content and facilitates Violaxanthin synthesis in *Arabidopsis thaliana*, further proving that *Pj*PSY1 overexpression enhances carotenoid biosynthesis in the fruits of *Panax japonicus*, demonstrating a positive correlation between them.

## 4. Discussion

Phytocarotenoids are widely found in nature and represent an important class of natural pigments in plants [17]. Existing studies have shown that the Phytoene synthase (PSY) gene serves as the first key enzyme gene in the carotenoid biosynthesis pathway. It has been verified that this gene facilitates carotenoid synthesis in various plants, including *Malus pumila* [18], *Solanum lycopersicum* [19], and *Zea mays* [20]. In this study, the *Pj*PSY1 gene was cloned from the fruit of *Panax japonicus* using RT-PCR, yielding an open reading frame of 1269 bp that encodes 423 amino acids, classified as a hydrophilic protein. This finding aligns with the results of the Phytoene synthase gene cloned from Tagetes erecta [12]. *Pj*PSY1 belongs to the superfamily of trans-isoprenyl diphosphate synthases (IPPS) and class I terpene cyclases, capable of synthesizing numerous precursors for carotenoid end products [21]. The *Pj*PSY1 gene is localized in chloroplasts, consistent with the subcellular localization of PSY genes in other reported species [22]. Phylogenetic analysis revealed that *Pj*PSY1 of *Panax japonicus* is most closely related to *Dc*PSY from *Daucus carota*, suggesting functional similarities. Studies have shown that *Dc*PSY plays a crucial role in carotenoid biosynthesis, leading to the hypothesis that *Pj*PSY1 also promotes carotenoid synthesis [23]. To verify this function, the pBI121-*Pj*PSY1 overexpression vector was constructed and transfected into *Arabidopsis thaliana* using the flower dip method. The carotenoid monomer content of the positive *Arabidopsis thaliana* plants was compared to that of the wild type, confirming that *Pj*PSY1 overexpression can enhance carotenoid synthesis in the fruits of *Panax japonicus*. Furthermore, this study found that *Pj*PSY1 could promote the synthesis of Violaxanthin, a lutein component, in *Arabidopsis thaliana*, which helps protect plants from strong light damage as well as helping them to utilize excess light energy and improve light energy efficiency [24]. From previous studies, which mainly focused on the PSY gene to promote carotenoid synthesis [25], we found that *Pj*PSY1 is involved in securing the supply of key pigments required for photosynthesis and maintaining the photosynthetic efficiency of plants under high light and in other environments by promoting Violaxanthin synthesis. At the same time, Violaxanthin is a precursor of the phytohormone abscisic acid (ABA) [26], which plays a crucial role in plant development and stress response.

## 5. Conclusions

We identified the *Pj*PSY gene, which plays a key role in carotenoid synthesis, in *Panax japonicus*. The expression level of *Pj*PSY was identified by heterologous expression in *Arabidopsis thaliana*, which directly affects the accumulation of carotenoids and provides a basis for the subsequent optimization of metabolic engineering. The PSY gene database was enriched to provide a basis for further analysis of the carotenoid synthesis mechanism in *Panax japonicus*.

## Data Availability

The original contributions presented in this study are included in the article. Further inquiries can be directed to the corresponding author.
